# Neutrophil Extracellular Traps and Microcrystals

**DOI:** 10.1155/2017/2896380

**Published:** 2017-03-07

**Authors:** Balázs Rada

**Affiliations:** College of Veterinary Medicine, Department of Infectious Diseases, The University of Georgia, Athens, GA, USA

## Abstract

Neutrophil extracellular traps represent a fascinating mechanism by which PMNs entrap extracellular microbes. The primary purpose of this innate immune mechanism is thought to localize the infection at an early stage. Interestingly, the ability of different microcrystals to induce NET formation has been recently described. Microcrystals are insoluble crystals with a size of 1–100 micrometers that have different composition and shape. Microcrystals have it in common that they irritate phagocytes including PMNs and typically trigger an inflammatory response. This review is the first to summarize observations with regard to PMN activation and NET release induced by microcrystals. Gout-causing monosodium urate crystals, pseudogout-causing calcium pyrophosphate dehydrate crystals, cholesterol crystals associated with atherosclerosis, silicosis-causing silica crystals, and adjuvant alum crystals are discussed.

## 1. Neutrophil Extracellular Traps

**Figure 1:**
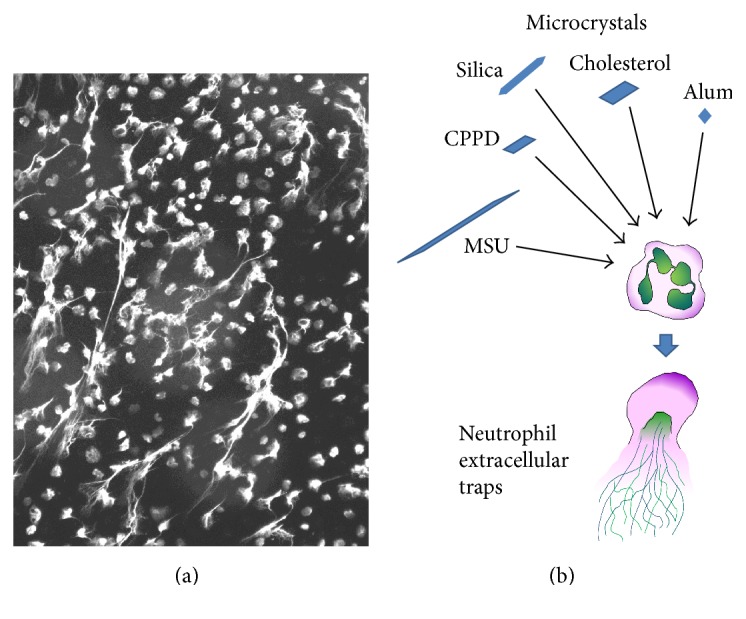
Neutrophil extracellular traps. (a) This fluorescent image depicts NETs released from human PMNs following CPPD crystal stimulation (50 *μ*g/mL, 3 hrs, unpublished data). PMN DNA was stained by DAPI and the color was artificially turned into white for better visibility. (b) Scheme demonstrating different types of microcrystals that were documented to release DNA from PMNs.

NET formation is a breathtaking mechanism by which neutrophil granulocytes (PMNs) trap extracellular pathogens (Figure 1) [1]. This innate immune mechanism involves remarkable cellular and molecular changes in PMNs. The membranes of granules and the nucleus dissolve, and the cytosolic and nuclear contents fuse [2]. The tightly packed, multilobulated nucleus of stimulated PMNs decondenses and will be released in the extracellular space (Figure 1) [1, 2]. The released DNA is associated with a variety of proteins, mainly histones and primary granule components. In fact, protein-DNA complexes have been used to define NET-derived extracellular DNA (ecDNA) and to distinguish it from DNA released from PMNs by other mechanisms [3, 4]. In addition to PMNs, eosinophil granulocytes, mast cells, and macrophages have also been shown to release extracellular traps, and ET formation has been documented in several species including humans [5–7]. Although the signaling steps in PMNs leading to NET formation remain largely unknown, a few steps are accepted. The NADPH oxidase was identified first as an enzyme essential for the extrusion of NETs [2]. Later on, the critical contributions of myeloperoxidase and neutrophil elastase were also revealed [8, 9]. A milestone in the process of understanding the mechanism of NET formation was the discovery that citrullination of histones by peptidylarginine deiminase 4 (PAD4) is also crucial [10–12]. Although these molecules are important in mediating NET formation, more recent results indicate that their contribution to the process is likely stimulus-, species-, and context-dependent [13–16]. These observations are also in line with the notion that the complicated process of NET formation is unlikely mediated by a single signaling pathway but rather by a complex network of molecular and cellular events. A wide range of stimuli has been described that stimulate NET release in PMNs including whole microbes (bacteria, viruses, fungi, and parasites), soluble molecules (microbial and host), and microcrystals of different origin [17, 18]. Trapping microorganisms is definitely a major function of NETs but might not be the only one. Considering the variety of agents triggering NETs under sterile inflammatory conditions including microcrystals discussed here, it is likely that NETs play a main role in the general inflammatory cascade, no matter what the stimulus. A novel role for NETs in limiting inflammation has already been proposed in gout, for instance [19]. Future research needs to clarify their exact physiological role, mechanism, and regulation. Microcrystals represent a unique set of NET-inducing stimuli (Figure 1) since they are particulate, can be phagocytosed, and form under different pathological conditions. In this review current knowledge on microcrystal-induced formation of NETs is summarized.

## 2. Monosodium Urate Crystals (MSU)

MSU crystals are the causative agents of the autoinflammatory condition, gout [21]. MSU crystals are negatively birefringent, needle-shaped, and generally 5–25 *μ*m (sometimes 100 *μ*m) in length [22, 23]. Uric acid is a degradation product of nucleic acid metabolism and crystallizes in the joints of gout patients in the form of needle-shaped crystals [21]. MSU crystals irritate the innate immune system including macrophages and PMNs leading to acute, painful attacks and chronic joint destruction [21, 24]. MSU crystal-induced PMN activation is a critical step in this inflammatory cascade and understanding its mechanism is crucial to developing novel anti-inflammatory therapies for gout.

PMNs attempt to phagocytose MSU crystals and produce reactive oxygen species (ROS) by the NADPH oxidase in response to them [33–35]. The first observation that MSU crystals induce NET release in PMNs was made by Mitroulis et al. showing that autophagy, PI3K signaling, and endosomal acidification are required for NET formation by MSU crystals [25]. The authors also described that gout synovial cells and peripheral PMNs of gout patients spontaneously release NETs, and gout synovial fluid and gout serum promote NET formation of PMNs obtained from healthy volunteers [25]. This observation was further expanded by Schorn et al. reporting that histones colocalize with DNA in MSU crystal-elicited NETs, and not only PMNs, but also basophil and eosinophil granulocytes also release NETs in response to MSU crystals [7]. They proposed that NETs immobilize the crystals, similarly how NETs would entrap bacteria [26]. The biological relevance of this finding was characterized in the landmark paper written by Schauer et al. suggesting that MSU crystal-induced formation of aggregated NETs (aggNETs) limits inflammation [19]. The high concentration of PMN proteases found in aggNETs was proposed to degrade several proinflammatory cytokines and put an end to recruitment of new leukocytes [19]. The authors showed that aggNETs formed in vitro and in vivo strongly reduced the amount of detectable proinflammatory cytokines [19]. They also found that mice deficient in the NADPH oxidase and incapable of making NETs developed an exacerbated, prolonged, chronic inflammation in contrast to control mice with normal NET-forming ability that had a restricted inflammatory response [19]. This phenomenon could be reversed by adoptively transferring aggNETs into NETosis-deficient mice [19].

**Figure 2:**
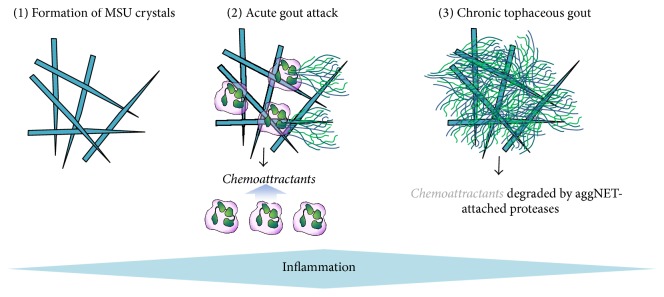
The proposed role of PMNs in the immunopathogenesis of gout. Phase (1) shows the deposition of needle-shaped MSU crystals. Phase (2) depicts PMNs phagocytosing crystals and releasing chemoattractants and NETs. Phase (3) shows the formation of aggregated NETs (aggNET) that provide the structural basis of gouty tophi and contain high concentration of PMN proteases degrading PMN chemoattractants.

Based on this study, the following role of NETs in gout pathogenesis has been proposed (Figure 2) [36]. First, PMNs recruited in large numbers to the joints of gout patients following inflammasome activation encounter MSU crystals (Figure 2) [36]. Activation of PMNs is accompanied with inflammation-associated pain in acute gout [36]. Whether NETs contribute to this phase of gout attack remains to be elucidated but is likely since by forming NETs PMNs also release their dangerous granule content. Second, at high PMN densities present at later stages of acute attacks, NETs form aggNETs that degrade proinflammatory cytokines and densely pack crystals to stop inflammation (Figure 2) [36]. AggNETs were proposed to form the basis for gouty tophi [19], a long-described white material that typically appears at the end of acute attacks and is characteristic for the chronic phase of gout (Figure 2) [19, 37]. Overall, aggNET formation was proposed to stop the acute inflammatory response at the expense of forming tophi that have been associated with symptoms of chronic gout [19, 36]. Recently, some of these data have been challenged [38]. Future studies are required to work out all the details of this mechanism [39]. Whether the general PMN-mediated inflammatory cascade has a built-in breaking mechanism identical or similar to the one described in gout remains an exciting, open question.

Despite its proposed novel role in gout pathogenesis, less is known about the cellular and molecular mechanism and regulation of MSU crystal-elicited NET formation. The requirement of a functional NADPH oxidase for MSU crystal-evoked NET release has been shown [19]. PMNs of patients suffering from chronic granulomatous disease (CGD) are unable to release NETs in response to PMA, bacteria [2], and MSU crystals [19]. NADPH oxidase deficient murine PMNs stimulated with MSU crystals do not release NETs and aggNETs, neither in vitro, nor in vivo [19]. Interestingly, soluble uric acid, not its crystallized form, stimulates NET release in an NADPH oxidase-independent manner [40]. These results indicate that NET release in gout must be complex, and multiple mechanisms could be responsible for mediating it. Authophagy has also been proposed to mediate NET formation induced by MSU crystals and other stimuli [25, 41, 42]. In a study by Desai et al. the involvement of RIPK1-RIPK3-MLKL signaling has been proposed in MSU crystal- and PMA-induced NET formation suggesting that NETosis is actually a PMN-specific necroptotic pathway [27]. This has been challenged by Amini et al. showing that NET release can occur independently of RIP3K and MLKL signaling, in response to PMA at least [43]. Thus, the relationship between NET formation and PMN necroptosis remains to be studied in more detail. In a recent study performed by Sil et al., we found that PMNs need to attempt to phagocytose MSU crystals in order to perform subsequent NET release and to form aggNETs [23]. PMNs do not really phagocytose MSU crystals since most of the crystals are far longer than PMNs themselves [23]. Our data indicated that only a small fraction of PMNs engaged in attempting MSU crystal phagocytosis but NET-releasing PMNs were all associated with MSU crystals [23]. This let us conclude that MSU crystal phagocytosis is a prerequisite for NET formation [23]. We proposed the involvement of the purinergic P2Y6 receptor in this mechanism based on a strong reduction of MSU crystal-induced NET release by general purinergic receptor inhibitors and the P2Y6-specific inhibitor MRS2578 [23]. Interestingly, exonucleotides alone failed to induce NET release in human PMNs [23]. On the other hand, MRS2578 reduced MSU crystal-stimulated ROS production, cytokine release, and PMN migration suggesting the involvement of these steps in MSU crystal-promoted NET extrusion [23]. In a separate study we revealed that interleukin-1*β* (IL-1*β*) derived from macrophages enhances NET release triggered by MSU crystals [28]. IL-1*β* promotes NET formation but NETs degrade cytokines including IL-1*β*; what could be the relevance of these two, opposite mechanisms in vivo in acute gout? They are most likely separated in time during the inflammatory process. While, at the early stage of gout flares, IL-1*β* drives inflammation, PMN recruitment and activation (proinflammatory segment), NETs become important later when sufficient levels accumulated capable of aggNET formation and cytokine degradation (anti-inflammatory phase). The details of this complex in vivo mechanism are, however, not well-understood. We and others also showed that anakinra, a potent IL-1 receptor antagonist, and antibodies neutralizing IL-1*β* inhibit the NETosis-enhancing effect of macrophages and gout synovial fluid [25, 28]. These results add a novel mechanism by which anakinra works and describe IL-1*β* as a potentiator of NET formation linking two significant arms of the inflammatory cascade in gout, inflammasome activation in macrophages, and NET formation in PMNs. A recent work by Pieterse et al. emphasized the critical role of phagocytes engulfing small urate microaggregates (SMA) in hyperuricemic blood [44]. These SMAs form first before they grow into long, needle-shaped MSU crystals that are known to trigger NET release [44]. Phagocytes take up SMAs and prevent the formation of MSU crystals and NETs in the circulation [44].

## 3. Calcium Pyrophosphate Dehydrate Crystals (CPPD)

Pseudogout is a condition similar to gout also characterized by periodic acute joint attacks that potentially turn into a chronic disease. Pseudogout is, however, caused by a different inflammatory microcrystal, calcium pyrophosphate dihydrate (CPPD) crystals [45]. CPPD crystals are typically shorter than MSU crystals and have a more rhomboid shape in contrast to the needle-like form of MSU crystals [29]. The pathomechanism of pseudogout is less studied than that of gout but PMN accumulation and its coincidence with painful attacks are also characteristic [46]. In a paper by Pang et al. we described robust in vitro NET formation of human PMNs in response to CPPD crystals [29]. CPPD crystals represent a much stronger NET-inducing signal for PMNs than MSU crystals [23, 28, 29]. We found that PMNs phagocytose CPPD crystals that is also a requirement for CPPD crystal-triggered NET release [29]. PMN nuclei underwent the same, characteristic morphological changes following CPPD crystal stimulation [29] as after PMA challenge [47]. The nucleus of PMNs undergoing NET formation first loses its segmented nature and lobi [1, 2, 29, 47]. Next, the nuclear material decondenses leading to the appearance of diffuse NETs followed by the formation of full-blown spread NETs [29, 47]. NADPH oxidase activity was not needed for CPPD crystal-elicited extrusion of NETs (Table 1) while it has been reported to be essential for MSU crystal-stimulated NET formation [19]. The NET-inducing ability of CPPD crystals required the activity of the heat shock protein 90, PI3K, and CXCR2 [29]. These results indicate that while both crystals induce NET release in human PMNs, different signaling pathways might be responsible for mediating the process.

## 4. Alum

**Figure 3:**
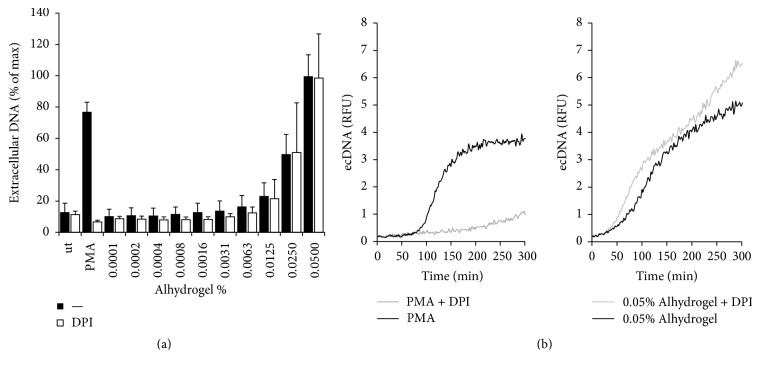
PMNs release extracellular DNA in response to Alhydrogel in vitro. Human PMNs seeded on a 96-well black microplate were incubated for 30 minutes in the presence or absence of 10 *μ*M DPI prior stimulation with increasing doses of commercially available Alhydrogel (InvivoGen, cat#: vac-alu-50) or 100 nM PMA. Increase in fluorescence due to extracellular DNA (ecDNA) release was measured in presence of 10 *μ*M Sytox Orange DNA-binding dye for 5 hours with a microplate fluorimeter. DNA release is presented as either relative fluorescence units (RFU) or percentage of maximal DNA released achieved by saponin treatment [4, 20]. (a) Summary of three independent experiments using PMNs obtained from independent human donors. Mean +/− SEM. (b) Representative kinetics of fluorescence results (*n* = 3). Ut, untreated; PMA, phorbol myristate acetate.

Alum is the most successful vaccine adjuvant used in the history of human medicine [48]; its exact mechanism of action remains, however, largely unknown to this day. Alum is composed of microcrystals and is thought primarily to enhance the efficacy of vaccines by increasing antigen phagocytosis by antigen presenting cells and by serving as an antigen depot [49]. Although PMNs are not the first cell type that comes to our mind when thinking of the mechanism of action of adjuvants, recent publications suggest that PMNs could play an important role in mediating or fine-tuning the immune response in the presence of adjuvants [50–52]. PMNs are rapidly recruited to the site of vaccination in large numbers; therefore, studying their interaction with adjuvants is clinically relevant since they could significantly alter the immune response at this early stage. PMNs have already been shown to release fibrin-like extracellular traps in the presence of aluminium adjuvants in vivo in mice [53]. No study has been performed though on how human PMNs interact with alum crystals in vitro. We therefore isolated human PMNs from the peripheral blood of healthy volunteers according to previously described protocols [20, 29] and stimulated them with aluminium adjuvant (Alhydrogel, InvivoGen) to detect extracellular DNA release using the DNA-binding, membrane-impermeable dye, Sytox Orange [4]. As our previously unpublished data show in Figure 3, PMNs responded to increasing concentrations of Alhydrogel with extracellular DNA release. This alum-induced DNA release was independent of reactive oxygen species production since the NADPH oxidase inhibitor diphenyleneiodonium (DPI) was without any effect (Figure 3). These data suggest that PMNs release their DNA upon alum crystal exposure. Future experiments are required to reveal the exact nature of this cell death mechanism.

## 5. Cholesterol Crystals

The important role of IL-1*β* in the pathogenesis of atherosclerosis has been well known but the mechanism by which macrophages release this cytokine remained poorly understood. Warnatsch et al. demonstrated recently that PMNs and NETs are crucial for both priming and stimulating macrophages to secrete IL-1*β* that will recruit additional PMNs to the atherosclerotic lesions [30]. PMNs have been previously implicated in the pathogenesis of atherosclerosis but their exact role has been unclear [54, 55]. These researchers showed that cholesterol crystals induce NET release in vitro in human PMNs in a concentration range that also activates the inflammasome [30]. Cholesterol crystals stimulated ROS production in PMNs and NET formation was blocked by the NADPH oxidase inhibitor DPI (Table 1) [30]. Neutrophil elastase translocated to the nucleus during cholesterol crystal-triggered NET formation but the PAD4 inhibitor Cl-amidine was without any effect [30]. NETs were also detected in vivo in lesions but were entirely absent in ApoE/PR3/NE-deficient mice lacking apolipoprotein E, neutrophil elastase, and proteinase 3 [30]. NET-deficient animals on high fat diet exhibited a reduced lesion size after 8 weeks proposing that NETs promote lesion formation in atherosclerosis [30]. NETs were required for enhanced cytokine production by macrophages in presence of cholesterol crystals that activated Th17 cells and amplified leukocyte recruitment [30]. The authors concluded that danger signals fuel sterile inflammation in atherosclerosis via PMNs [30].

## 6. Silica Crystals

Chronic exposure to silica crystals leads to pulmonary silicosis or chronic obstructive pulmonary disease and also relates to vasculitis or chronic renal failure [56, 57]. Silica crystals activate the inflammasome and can be phagocytosed by immune cells including PMNs [58]. NETs have also been associated with glomerulonephritis and small vessel vasculitis as the source of antineutrophilic cytoplasmic antibodies [59, 60]. Although silica crystal stimulation of murine PMNs leads to ROS release, the in vivo relevance of this finding has not been established yet [61]. Brinkmann et al. described extracellular DNA release in human PMNs challenged with different doses of silica crystals suggesting that silica crystal-promoted NETs could play an important role in the establishment of lung disease [31]. PMNs are known to be recruited in large numbers to the lungs in silicosis animal models and human patients [62–64]. While silica crystal-stimulated DNA release from PMNs was comparable to that induced by MSU crystals [31], eosinophils did not release ETs in the presence of silica crystals [7].

## 7. Conclusion

Despite their different origin and structure, microcrystals activate PMNs leading to an inflammatory response. PMNs attempt to engulf microcrystals that is required for launching their effector responses including ROS production and NET release. Although a young and specific field, PMN-microcrystal interactions are clinically relevant to study due to their involvement in diverse biological processes ranging from disease pathologies of sterile autoinflammatory and infectious diseases to vaccination.

## Figures and Tables

**Table 1:** Microcrystals that trigger NET formation.

Crystal name	Clinical relevance	Requirement of the following				References
		NADPH oxidase	PAD4	MPO	NE	
Monosodium urate (MSU)	Gout	Yes	?	?	No	[7, 19, 23, 25–28]
Calcium pyrophosphate dehydrate crystals (CPPD)	Pseudogout	No	?	?	?	[29]
Cholesterol crystals	Atherosclerosis	Yes	No	?	Yes	[30]
Silica crystals	Silicosis	?	?	?	?	[31]
PMA (in comparison)	—	Yes	?	Yes	Yes	[2, 9, 32]
